# First Discovery of *Vespa velutina nigrithorax* du Buysson (Hymenoptera: Vespidae), an Invasive Hornet in the Feces of the Yellow-Throated Marten in South Korea

**DOI:** 10.3390/insects12040296

**Published:** 2021-03-29

**Authors:** Chang-Jun Kim, Moon Bo Choi

**Affiliations:** 1 Division of Forest Biodiversity, Korea National Arboretum, Pocheon 11186, Korea; changjunkim@korea.kr; 2 School of Applied Biosciences, College of Agriculture & Life Sciences, Kyungpook National University, Daegu 41566, Korea; 3 Institute of Agricultural Science and Technology, Kyungpook National University, Daegu 41566, Korea

**Keywords:** invasive alien species, feeding behavior, hornet, mammal, *Martes flavigula*, predator, wasp

## Abstract

**Simple Summary:**

Invasive species can have serious economic and ecological effects. Biological controls are a way to reduce damage from invasive species. We collected 22 fecal samples from yellow-throated martens, which often prey on wasps in late autumn, from Mt. Onggangsan in Cheongdo, South Korea, to confirm the predation of the invasive alien hornet, *Vespa velutina nigrithorax*. Hornet debris was found in three samples, along with two native wasp species. The hornets were identified as one queen, four males, one female, and one individual whose caste was unclear. Therefore, because reproductive individuals were preyed upon, it is likely that yellow-throated marten predation could potentially be used for the biological control of invasive alien hornets.

**Abstract:**

Yellow-throated martens (YTMs) are omnivores that often prey on wasps in late autumn in Korea. However, to the best of our knowledge, predation of the invasive alien species *Vespa velutina nigrithorax* (VVN) has not previously been investigated. In this study, YTM feces were collected and analyzed from Mt. Onggangsan, Sinwon-ri, Cheongdo, South Korea, where VVN density was high and YTMs were active. Surveys were conducted three times between October and December 2019, during which a total of 22 samples were collected. Debris from VVN was found in three samples, along with evidence of two indigenous wasps, *Vespa crabro* and *Vespula koreensis*. The VVN remains were identified as one queen, four males, one female, and one individual whose caste was unclear. Martens prey on wasps, owing to a sudden decrease in plant food sources from late autumn to early winter, mostly eating males and new queens attempting to mate. If VVN reproduction is prevented or disturbed by YTM predation, there may be potential biological control effects in areas with high VVN density. Further studies should be conducted to verify whether there is a practical biological control effect.

## 1. Introduction

Invasive alien species (IAS) reduce biodiversity and have economic and ecological impacts as they can spread widely because of climate change and global trade [1,2]. Biological controls are widely used to prevent damage from IAS [3]. Biological control using natural enemies is highly efficient and is widely used in crop systems and forest ecosystems to replace the existing chemical controls in an eco-friendly manner [4,5,6,7]. In particular, biological controls show a better effect through a customized approach depending on the characteristics of each IAS [8]. Social insects such as wasps, ants, and bees are among the most serious IASs; large colonies may cause serious ecological disturbances, such as species displacement, and dominate competing indigenous species in invaded areas [9,10,11]. Social wasps are the top predators in insect communities. However, social wasps have various parasitic and predatory natural enemies that control their abundance and density within an ecosystem [12]. 

The common parasitoid, *Xenos* spp. (Stylopidae: Strepsiptera [13,14,15], along with *Conops* spp. (Conopidae: Diptera) [16], *Pheromermis* spp. (Mermithidae: Nematoda), and *Sphaerularia* spp. (Sphaerulariidae: Nematoda) [17,18] are endoparasites in adult wasps. Parasitoids living in wasp colonies include Ichneumonidae (*Latibulus* spp. and *Sphecophaga* spp.) [19,20], Trigonalyidae (*Bareogonalos* spp. and *Metoecus* spp.) [21,22,23], and Eulophidae (*Elasmus* spp.) [14,24] are known in Hymenoptera. *Volucella* spp. of Syrphidae [25] are known in Diptera, and *Pyralis* spp. and *Hypsopygia* spp. of Pyralidae [26] are known in Lepidoptera. 

The majority of wasp predators are birds and mammals. Predatory birds, including honey buzzards (*Pernis apivorus*), European bee-eaters (*Merops apiaster*), Eurasian magpies (*Pica pica*), great tits (*Parus major*), and Eurasian nuthatches (*Sitta europaea*) [27,28] and mammals, including badgers (*Meles meles*), bears (*Ursus americanus*), racoons (*Procyon lotor*), skunks (*Mephitis* sp.), and martens (*Martes flavigula*, *Martes americana* and *Martes martes*) [29,30,31,32,33,34,35], are known to attack small and medium-sized wasp nests in order to prey on individuals. 

The IAS *Vespa velutina nigrithorax* (VVN) first invaded the Korean Peninsula in 2003 from China and has since spread nationwide [10,36,37,38,39]. In 2014, it spread to Japan [40]. In Europe, after the first invasion into France in 2004 [41], VVN spread to approximately 10 European countries at a rapid rate [42,43]. VVN is a harmful invasive species that has more complex impacts than many other invasive species, including economic and public health impacts, as well as ecological disturbances [44,45,46,47].

In Europe, VVN is being removed, controlled, and managed by attractant traps [48], harmonic radars [49], radio telemetry [50], and other methods [51]. Various methods such as trapping attractants, netting, and capturing queens have been implemented in Korea; however, to date, these methods have been inefficient [52]. However, to the best of our knowledge, no studies have been conducted on the biological control of VVN. A parasitoid and parasite have recently been discovered in Europe: initially, the conopid fly, *Conops vesicularis* (Conopidae, Diptera), whose larvae are endoparasitoids of bees and wasps [16], followed by *Pheromermis vesparum* (Mermithidae, Nematoda), which was found as endoparasite of arthropods [18]. The honey buzzard, *Pernis apivorus*, was recorded as the first predator to attack VVN nests [28]. 

In Korea, *Xenos* sp. (Strepsiptera: Stylopidae) is the only reported ectoparasitoid of *Vespa* species [13]. Asiatic black bears [53] and yellow-throated martens (YTM; *Martes flavigula* [34]), which are not only designated as endangered species (Class I and II, respectively) in South Korea [54], but are also on the Red List of the International Union for Conservation of Nature (IUCN) [55], are known as the only predators of the *Vespa* species. However, there is no known predation of VVN. 

Therefore, the purpose of this study was to confirm whether YTMs inhabiting the forest area of South Korea preyed on VVN. An area with high VVN density and frequent appearance of YTMs was selected [38,56], and YTM feces were investigated in and around the location where they were observed in the area, to confirm the predation of VVN by YTM.

## 2. Materials and Methods

**Figure 1:**
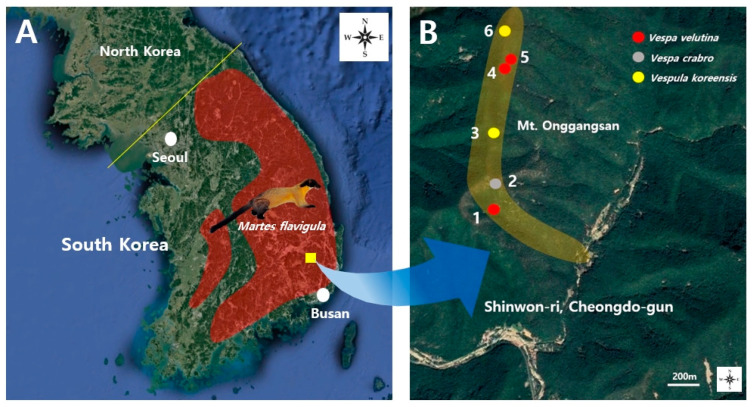
Distribution area of the yellow-throated marten (YTM) in South Korea and the survey area. (**A**) YTM habitat area distributed along the southeastern mountainous area of South Korea (red area) (modified from [59]). (**B**) The location of YTM feces where wasp debris was detected in the area of Mt. Onggangsan, Sinwon-ri, Cheongdo-gun. Yellow area: survey area (see Table 1 for collection points).

This survey was conducted on Mt. Onggangsan, Sinwon-ri, Cheongdo-gun, Korea, where VVN density was relatively high (Figure 1B, Table 1) and YTM activity has been recorded in South Korea (Figure 1A). According to Choi et al. [34], YTMs prey on wasps, mainly between late autumn and early winter. Therefore, this survey was conducted three times (i.e., once a month) between October and December 2019. YTM feces are approximately 6–12 cm long and 2–2.5 cm thick and are characteristically excreted on small rocks or fallen trees about 1 m from trails [56]. Siberian weasel (*Mustela sibirica*: Mustelidae) feces of similar shape are clearly distinguished because they are less than 6 cm long and 1 cm thick. Nevertheless, feces were confirmed as YTM by two mammologists (Dr. DG Woo of National Institute of Ecology and Mr. SG Lee of Korea Wildlife Ecology Institute). Therefore, using these characteristics, YTM feces were collected by searching for approximately 2.1 km, starting from the entrance of the trail to the top of Mt. Onggangsan, approximately 10 m to the left and right of the trail (Table 1, Figure 1B). The collected feces were placed in a vinyl pack and sealed, and the collection date and coordinates were recorded. The samples were then transported to the laboratory of Kyungpook National University and stored in the refrigerator to prevent unpleasant odors and deterioration. The samples were immersed in water according to the standard method [57] to dissolve the contents. They were then filtered with a mesh sheet (5 mm, 1 mm) and dried for 5 h at 50 °C in a dry oven. The dried samples were separated from wasp debris using a microscope (Model: Leica Microsystems SG/S9D) and the wasp debris was identified as follows, based on the methods of Archer [58] and Kim et al. [36]. 

The distinction between female and male wasps was based on the shape of the clypeus on the head and the shape of the last segment of the abdomen. The lateral angles of the female clypeus are more developed than that of the male. The tip of the last segment of the abdomen of the female is sharp, where the last segment of the male is blunt. In addition, there is a difference in size between the queen and the worker VVN, and the caste was estimated by the width of the head (the average head width of queens (n = 5): 5.98 ± 0.03 mm, collected in May 2019; the average head width of workers (n = 5): 4.88 ± 0.11 mm, collected in October 2019; all samples were collected in Daegu, close to Cheongdo-gun). In *Vespula koreensis*, the queen and worker are markedly different in respect to the abdominal pattern [58], and the abdominal pattern was identified in the debris that remained intact. However, when the distinction between a worker and queen was ambiguous, the specimen was designated as female, and when sex and caste were both unclear, the specimen was specified as a wasp. The number of individuals was calculated based on the number of heads, thoraxes, and abdomens.

## 3. Results

**Figure 2:**
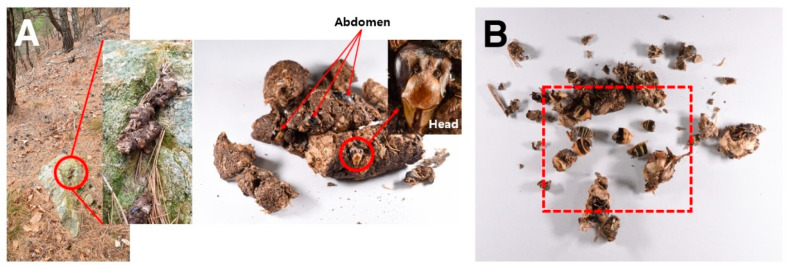
Wasp debris detected in yellow-throated marten (YTM) feces at Mt. Onggangsan. (**A**) Detection of debris of the male head and abdomen of the invasive alien hornet, *Vespa velutina nigrithorax* (VVN), in YTM feces (feces collection area 1). (**B**) Queen and male debris of *Vespula koreensis* detected in YTM feces (feces collection area 3).

A total of 22 YTM fecal samples were collected from the survey areas, of which wasp debris was found in six samples (Table 1, Figure 1). In the first survey (October 23), wasp debris was found in two (No. 2 and 6 collection areas) out of the eight fecal samples collected, and they were identified as the indigenous wasps, *Vespa crabro* (two females) and *Vespula koreensis* (three queens and five males) (Table 1, Figure 1B). On November 13 (the second survey), wasp debris was found in three (No. 1, 3, and 4 collection areas) out of a total of nine fecal samples (Table 1, Figure 1B). One female and four male VVN debris were first detected in two fecal samples (Figure 2A), and the remaining debris was identified as that of *Vespula koreensis* (four queens and two males) (Table 1, Figure 2B). In the final survey on December 4, wasp debris was found in two (No. 5 collection area) out of a total of five samples, in which one queen VVN (measured head width: 5.96 mm) and one unidentified VVN wasp were detected (Table 1, Figure 1B). Therefore, it was confirmed that YTMs prey on the VVN queen (females) or males between late autumn and early winter, as a VVN queen (female) and males were found in a total of three fecal samples.

## 4. Discussion

Approximately 3000 YTMs are currently estimated to inhabit South Korea [60]. YTMs are omnivorous and highly fluid, depending on the available food resources and the season [61]. In general, the diet of YTMs is composed of approximately 60% plants (such as fruit), followed by approximately 35% mammals (e.g., wild boar, elk, and rats), and approximately 5% insects [56]. In particular, >80% of the insects that they eat are social wasps, mostly found between late autumn and early winter [34]. This is because the defense behavior of numerous wasp workers in the mature nest is strong in the summer, and, therefore, it is difficult for YTMs to directly access the nest; additionally, other foods are sufficient so YTMs rarely feed on wasps during summer [34]. However, wasps are a useful food resource that can provide a protein source for YTMs because males without poison and new queens with very low aggression appear in late autumn when the other food sources rapidly reduce [34]. Some martens in Europe and North America show similar habits, such as consuming yellowjackets in autumn [35,62,63]. Therefore, this predatory habit of YTMs appears to have a potential to contribute to the biological control of VVN. Of course, YTMs may accidentally eat aged workers and queens that die in late autumn, or ingest dead individuals left in extinct nests, but YTMs are known to mainly hunt living organisms due to their very aggressive hunting habits [56]. According to Woo [56], in the predation test following the marten’s attack on the beehive, the dead bees were not eaten at all, so the possibility is not great that dead wasps are eaten. 

Recently, biological control methods that effectively reduce the population density through reproductive blocking and disturbance of invasive species and pests have been widely applied [64,65,66]. There is a gradual increase in VVN density in South Korea; therefore, if the predation by YTMs is concentrated in the late autumn when VVN males and new queens mate, then there is a possibility that it will have some effect on the density reduction of VVN. While YTMs can prey on native wasps, which are non-target species as biocontrol agents. However, some native wasps and YTMs have established natural enemy-prey relationships for a long time [34], so the impact on the density of native wasps should be negligible. 

In addition, in the mountainous regions of north-central South Korea, the YTM density is high [56], while the VVN density is low [38], and therefore, VVN spread can be expected to be minimized or entirely prevented, thus leading to a reduction of ecological disturbances and biodiversity conservation. 

Wasp debris was also found in the feces of the Asiatic black bear (*Ursus thibetanus ussuricus*) in the area of Jirisan National Park in South Korea [67]. In South Korea, this bear used to be extinct; however, a restoration project was started in Jirisan National Park in 2004, and approximately 60 black bears now inhabit the area [59,68]. According to Jung et al. [53], 12.8–14.2% of the excrement of these bears was composed of insects such as bees, wasps, ants, and beetles, and most of these insect food sources appeared between October and November. Therefore, Asiatic black bears have similar feeding habits to those of YTM. As their distribution area gradually expands [68], their biological control of VVN is likely to be effective, although follow-up studies are needed to confirm this. Despite the YTM predation activity and the existing parasites, the possibility of dramatically reducing VVN density is very low. However, it is expected that an ecological balance will gradually be attained if the action of natural enemies continues along with the control activities by humans. In addition, in the present study, the presence or absence of YTM predation in a small local area was confirmed. Therefore, in the future, the effect of YTM predation on the reduction of VVN density can be verified. Detailed investigations should be conducted in areas with large YTM habitats (such as Jirisan National Park, Sobaeksan National Park, and mountainous areas in Gangwon-do).

## 5. Conclusions

As a result of analyzing the feces of YTMs, known as a predator of wasps in South Korea, debris of VVN and the indigenous wasps *Vespa crabro* and *Vespula koreensis* were found. YTMs appeared to prey on wasps mainly in late autumn to early winter as a protein source. In particular, YTMs preyed on new queens and males of VVN, providing clues for the biological control of this IAS. In the future, a more detailed investigation is needed on the control effect of VVN caused by marten predation.

## Figures and Tables

**Table 1:** Location information for the yellow-throated marten (YTM) fecal samples containing wasp debris and the identified species, caste, and number of individuals between October and December 2019.

Fecal Collection Area	Coordinates	Altitude (m)	Date of Collection	Identification	Sex and Caste	Number of Individuals	* Number of Fecal Samples
1	N35°40′52.06″ E129°00′03.62″	501	11/13	*Vespa velutina*	Female	1	1
					Male	2	
2	N35°40′56.22″ E129°00′03.76″	593	10/23	*Vespa crabro*	Female	2	1
3	N35°41′07.06″ E129°00′05.52″	583	11/13	*Vespula koreensis*	Queen	4	1
					Male	2	
4	N35°41′21.13″ E129°00′11.73″	716	11/13	*Vespa velutina*	Male	2	1
5	N35°41′24.50″ E129°00′14.40″	728	12/4	*Vespa velutina*	Queen	1	1
					Wasp	1	
6	N35°41′41.87″ E129°00′20.81″	722	10/23	*Vespula koreensis*	Queen	3	1
					Male	5	

* No wasp debris were found in the remaining 16 fecal samples.

## Data Availability

No additional data available.

## References

[1] Hulme P.E. (2009). Trade, transport and trouble: Managing invasive species pathways in an era of globalization. J. Appl. Ecol..

[2] Ziska L.H., Blumenthal D.M., Runion G.B., Hunt E.R., Diaz-Soltero H. (2011). Invasive species and climate change: An agronomic perspective. Clim. Chang..

[3] Heimpel G.E., Cock M.J. (2018). Shifting paradigms in the history of classical biological control. BioControl.

[4] Van Lenteren J.C., Gurr G., Wratten S. (2000). Success in biological control of arthropods by augmentation of natural enemies. Biological Control: Measures of Success.

[5] Van Lenteren J.C. (2012). The state of commercial augmentative biological control: Plenty of natural enemies, but a frustrating lack of uptake. BioControl.

[6] Landis D.A., Wratten S.D., Gurr G.M. (2000). Habitat management to conserve natural enemies of arthropod pests in agriculture. Annu. Rev. Entomol..

[7] Duan J.J., Bauer L.S., Abell K.J., Ulyshen M.D., Van Driesche R.G. (2015). Population dynamics of an invasive forest insect and associated natural enemies in the aftermath of invasion: Implications for biological control. J. Appl. Ecol..

[8] Wyckhuys K.A.G., Sasiprapa W., Taekul C., Kondo T. (2020). Unsung heroes: Fixing multifaceted sustainability challenges through insect biological control. Curr. Opin. Insect. Sci..

[9] Beggs J.R., Brockerhoff E.G., Corley J.C., Kenis M., Masciocchi M., Muller F., Rome Q., Villemant C. (2011). Ecological effects and management of invasive alien Vespidae. BioControl.

[10] Kwon O., Choi M.B. (2020). Interspecific hierarchies from aggressiveness and body size among the invasive alien hornet, *Vespa velutina nigrithorax*, and five native hornets in South Korea. PLoS ONE.

[11] Boulay R., Arnan X., Cerda X., Retana J. (2014). The ecological benefits of larger colony size may promote polygyny in ants. J. Evol. Biol..

[12] Huffaker C.B. (1971). Biological Control.

[13] Makino S., Kawashima M., Kosaka H. (2011). First record of occurrence of *Xenos moutoni* (Strepsiptera: Stylopidae), an important parasite of hornets (Hymenoptera: Vepidae: Vespa), in Korea. J. Asia Pac. Entomol..

[14] Mayorga-Ch D., Sarmiento C.E. (2020). Parasitoids of *Polistes myersi* Bequaert, 1934 (Vespidae, Polistinae). Sociobiology.

[15] Hrabar M., Danci A., McCann S., Schaefer P., Gries G. (2014). New findings on life history traits of *Xenos peckii* (Strepsiptera: Xenidae). Can. Entomol..

[16] Darrouzet E., Gevar J., Dupont S. (2015). A scientific note about a parasitoid that can parasitize the yellow-legged hornet, *Vespa velutina nigrithorax*, in Europe. Apidologie.

[17] Sayama K., Kosaka H., Makino S. (2007). The first record of infection and sterilization by the nematode Sphaerularia in hornets (Hymenoptera, Vespidae, Vespa). Insectes Soc..

[18] Villemant C., Zuccon D., Rome Q., Muller F., Poinar G.O., Justine J.L. (2015). Can parasites halt the invader? Mermithid nematodes parasitizing the yellow-legged Asian hornet in France. PeerJ.

[19] Oh S.H., An S.L., Lee J.W. (2012). Review of Korean Latibulus (Hymenoptera: Ichneumonidae: Cryptinae) and a key to the world species. Can. Entomol..

[20] Konishi K., Sayama K., Choi J.Y. (1997). Intraspecific color variation in *Sphecophaga vesparum* (Curtis) and subspecific status of the far eastern population (Hyemnoptera: Ichneumonidae). Jpn. J. Entomol..

[21] Kim C.J., Tan J.L., Lee B.W., Oh S.H., Choi M.B. (2020). Discovery of a trigonalid wasp, *Bareogonalos xibeidai* (Hymenoptera: Trigonalyidae), reared from nests of *Vespula koreensis koreensis* (Hymenoptera: Vespidae) in South Korea. J. Asia Pac. Biodivers..

[22] Yamane S. (2014). New taxa of the genus Bareogonalos from Asia with further information on the tribe Nomadinini (Hymenoptera, Trigonalidae). Halteres.

[23] Tan J.L., Van Achterberg C., Tan Q.Q., Zhao L.P. (2017). New species of Trigonalyidae (Hymenoptera) from NW China. ZooKeys.

[24] Kim I.K., Kwon O., Choi M.B. (2016). Two species of *Elasmus japonicus* Ashmead and *Elasmus polistis* Burks (Hymenoptera: Eulophidae) reared form nests of Polistes (Hymenoptera: Vespidae) in Korea. J. Asia Pac. Biodivers..

[25] Matsuura M., Yamane S. (1990). Biology of the Vespine Wasps.

[26] Martin S.J. (1992). Occurrence of the Pyralid Moth *Hypsopygia mauritialis* (Lepidoptera, Pyralidae) in the nests of *Vespa affinis* (Hymenoptera, Vespidae). Jpn. J. Entomol..

[27] Miyazaki M. (1981). Eagles Hawks Falcons.

[28] Macià F.X., Menchetti M., Corbella C., Grajera J., Vila R. (2019). Exploitation of the invasive Asian hornet *Vespa velutina* by the European honey buzzard *Pernis apivorus*. Bird Study.

[29] Fox-Wilson G. (1946). Factors affecting populations of social wasps, Vespula species, in England (Hymenoptera). Proc. R. Entomol. Soc. Lond. Ser. A Gen. Entomol..

[30] Trap-Lind I. (1962). Observations on a honey buzzard digging out a wasp’s nest. Br. Birds.

[31] Spradbery J.P. (1973). Wasps: An Account of the Biology and Natural History of Social and Solitary Wasps.

[32] MacDonald J.F. Comparative and adaptive aspects of vespine nest construction. Proceedings of the 8th International Congress IUSSI.

[33] Edwards R. (1980). Social Wasps: Their Biology and Control.

[34] Choi M.B., Woo D., Choi T.Y. (2015). Composition of the insect diet in feces of yellow-throated marten, *Martes flavigula*, in Jirisan National Park, South Korea. J. Ecol. Environ..

[35] Zielinski W.J., Spencer W.D., Barrett R.H. (1983). Relationship between food habits and activity patterns of pine martens. Mammal.

[36] Kim J.K., Choi M.B., Moon T.Y. (2006). Occurrence of *Vespa velutina Lepeletier* from Korea, and a revised key for Korean *Vespa* species (Hymenoptera: Vespidae). Entomolog. Res..

[37] Do Y., Kim J.B., Shim J.H., Kim C.J., Kwon O., Choi M.B. (2019). Quantitative analysis of research topics and public concern on *V. velutina* as invasive species in Asian and European countries. Entomolog. Res..

[38] Park J.J., Jung C. (2016). Risk prediction of the distribution of invasive hornet, *Vespa velutina nigrothorax* in Korea using CLIMEX model. J. Apicult..

[39] Granato A., Negrisolo E., Bonomi J., Zulian Z., Cappa F., Bortolotti L., Mutinelli F. (2019). Recent confirmation of a single haplotype in the Italian population of *Vespa velutina*. Biol. Invasions.

[40] Sakai Y., Takahashi J. (2014). Discovery of a worker of *Vespa velutina* (Hymenoptera: Vespidae) from Tsuhima Island, Japan (Japanese with English summary). Jpn. J. Appl. Entomol. Zool..

[41] Haxaire J., Bouguet J.P., Tamisier J.P. (2006). *Vespa velutina* Lepeletire, 1836, une redoutable novueauté pour la faune de France (Hymenoptera, Vespidae). Bull. Soc. Entomol. Fr..

[42] Husemann M., Sterr A., Maack S., Abraham R. (2020). The northernmost record of the Asian hornet *Vespa velutina nigrithorax* (Hymenoptera, Vespidae). Evol. Syst..

[43] Rome Q., Villemant C. Le Frelon Asiatique *Vespa velutina*. Inventaire National du Patrimoine Naturel—Muséum National d’Histoire Naturelle. http://frelonasiatique.mnhn.fr/home.

[44] Requier F., Rome Q., Chiron G., Decante D., Marion S., Menard M., Muller F., Villemant C., Henry M. (2019). Predation of the invasive Asian hornet affects foraging activity and survival probability of honey bees in Western Europe. J. Pest. Sci..

[45] Cini A., Cappa F., Petrocelli I., Pepiciello I., Bortolotti L., Cervo R. (2018). Competition between the native and the introduced hornets *Vespa crabro* and *Vespa velutina*: A comparison of potentially relevant life-history traits. Ecol. Entomol..

[46] Choi M.B., Kim T.G., Kwon O. (2019). Recent trends in wasp nest removal and Hymenoptera stings in South Korea. J. Medic. Entomol..

[47] Yamasaki K., Takahashi R., Harada R., Matsuo Y., Nakamura M., Takahashi J.I. (2019). Reproductive interference by alien hornet *Vespa velutina* threatens the native populations of *Vespa simillima* in Japan. Sci. Natur..

[48] Lioy S., Laurino D., Capello M., Romano A., Manino A., Porporato M. (2020). Effectiveness and selectiveness of traps and baits for catching the invasive hornet *Vespa velutina*. Insects.

[49] Milanesio D., Saccani M., Maggiora R., Laurino D., Porporato M. (2017). Recent upgrades of the harmonic radar for the tracking of the Asian yellow-legged hornet. Ecol. Evol..

[50] Kennedy P.J., Ford S.M., Poidatz J., Thiery D., Osborne J.L. (2018). Searching for nests of the invasive Asian hornet (*Vespa velutina*) using radio-telemetry. Comm. Biol..

[51] Turchi L., Derijard B. (2018). Options for the biological and physical control of *Vespa velutina nigrithorax* (Hym.: Vespidae) in Europe: A review. J. Appl. Entomol..

[52] Lee S.H., Lee S.J., Lee K.S., Heo J.H., Choi M.B., Yoon H.J., Hong I.P., Korea Apicultural Agriculture Cooperative, Korea Forest Service (2020). Korean Beekeeping Industry and Save Honeybees Movement in Korea.

[53] Jung D.H., Seomun H., Song D.J., Choi E.H., Lee S.H., Lee Y.H., Cho C.U., Song B.C., Yang D.H. (2016). Analysis of Asiatic black bear’s foods by using scats in the Jirisan National Park. Kor. J. Environ. Ecol..

[54] National Institute of Biological Resources. https://species.nibr.go.kr/home/mainHome.do?cont_link=011&subMenu=011013&contCd=011013001001.

[55] IUCN The IUCN Red List of Threatened Species. https://www.iucnredlist.org/.

[56] Woo D.G. (2014). A Study on Ecological Characteristics and Conservation of Yellow-Throated Marten (*Martes flavigula*) in Temperate Forests of Korea. Ph.D. Thesis.

[57] Jedrzejewska B., Jedrejewski W. (1998). Predation in Vertebrate Communities: The Bialowieza Primeval Forest as a Case Study.

[58] Archer M.E. (2012). Vespine Wasps of the World: Behaviour, Ecology & Taxonomy of the Vespinae.

[59] Lee S., Lee S., Song W., Lee M.J. (2017). Habitat potential mapping of marten (*Martes flavigula*) and leopard cat (*Prionailurus bengalensis*) in South Korea using artificial neural network machine learning. Appl. Sci..

[60] Choi T., Woo D., Yang B., Kim M., Lee S., Jeong W., Choi D. (2011). Management of Ecological Corridor to Conserve Wildlife Population (III).

[61] Zhou Y.B., Newman C., Xu W.T., Buesching C.D., Zalewski A., Kaneko Y., Macdonald D.W., Xie Z.Q. (2010). Biogeographical variation in the diet of Holarctic martens (genus Martes, Mammalia: Carnivora: Mustelidae): Adaptive foraging in generalists. J. Biogeogr..

[62] Slauson K.M., Zielinski W.J. (2017). Seasonal specialization in diet of the Humboldt marten (*Martes caurina humboldtensis*) in California and the importance of prey size. J. Mammalog..

[63] Pawlikowski T., Olszewski P., Piekarska-Boniecka H., Pawlikowski K. (2016). Diversity of social wasp communities (Hymenoptera: Polistinae and Vespinae) in the agricultural landscape of Central Poland. Acta Zool. Bulg..

[64] Prowse T.A.A., Cassey P., Ross J.V., Pfitzner C., Wittmann T.A., Thomas P. (2017). Dodging silver bullets: Good CRISPR gene-drive design is critical for eradicating exotic vertebrates. Proc. R. Soc. B.

[65] Alphey L., Benedict M., Bellini R., Clark G.G., Dame D.A., Service M.W., Dobson S.L. (2010). Sterile-insect methods for control of mosquito-borne diseases: An analysis. Vector Borne Zoonotic Dis..

[66] Zheng X., Zhang D., Li Y., Yang C., Wu Y., Liang X., Liang Y., Pan X., Linchao H., Sun Q. (2019). Incompatible and sterile insect techniques combined to eliminate mosquitoes. Nature.

[67] Seomun H. (2006). Analysis of Asiatic Black Bears’s Foods by Using Feces. Master’s Thesis.

[68] Kim J.J., Kim T.W., Choi J.Y., Park S.H., Han S.H., Lee S.H., Oh H.S. (2019). A case study of the habitat expansion of the Asiatic black bear (*Ursus thibetanus ussuricus*). Kor. J. Environ. Biol..

